# Patients infected by tuberculosis and human immunodeficiency virus facing their disease, their reactions to disease diagnosis and its implication about their families and communities, in Burkina Faso: a mixed focus group and cross sectional study

**DOI:** 10.1186/s13104-016-2183-3

**Published:** 2016-07-29

**Authors:** Ziemlé Clément Méda, Télesphore Somé, Issiaka Sombié, Daouda Maré, Donald E. Morisky, Yi-Ming Arthur Chen

**Affiliations:** 1 Ministry of Health, Ouagadougou, Burkina Faso; 2 International Health Program, Institute of Public Health, Bobo Dioulasso, Burkina Faso; 3 Muraz Research Center, Bobo Dioulasso, Burkina Faso; 4 Research Office of West African Health Organization (WAHO), Bobo Dioulasso, Burkina Faso; 5 National Institute of Health Sciences, Polytechnic University, Bobo Dioulasso, Burkina Faso; 6 Association Responsabilité-Espoir-Vie-Solidarité (REVS+), Bobo Dioulasso, Burkina Faso; 7 Department of Community Health Sciences, University of California Los Angeles (UCLA), School of Public Health, Los Angeles, USA; 8 Department of Microbiology and Institute of Medical Research, Kaohsiung Medical University, Kaohsiung City, Taiwan; 9 Center for Infectious Disease and Cancer Research (CICAR), Kaohsiung Medical University, Kaohsiung City, Taiwan

**Keywords:** Tuberculosis, HIV, Discrimination, Stigmatization, Healer, Gender, Preventive medicine, Global health

## Abstract

**Background:**

Patients facing tuberculosis (TB) and human immunodeficiency virus (HIV) infection receive particular care. Despite efforts in the care, misconceptions about TB and HIV still heavily impact patients, their families and communities. This situation severely limits achievement of TB and HIV programs goals. This study reports current situation of TB patients and patients living with HIV/AIDS (PLWHA) facing their disease and its implications, by comparing results from both qualitative and quantitative study design.

**Methods:**

Cross sectional study using mixed methods was used and excluded patients co-infected by TB and HIV. Focus group included 96 patients (6 patients per group) stratified by setting, disease profile and gender; from rural (Orodara Health District) and urban (Bobo Dioulasso) areas, all from *Hauts-Bassins* region in Burkina Faso. Quantitative study included 862 patients (309 TB patients and 553 PLWHA) attending TB and HIV care facilities in two main regions (*Hauts*-*Bassins and Centre*) of Burkina Faso.

**Results:**

A content analysis of reports found TB patients and PLWHA felt discriminated and stigmatized because of misconceptions with its aftermaths (rejection, emotional and financial problems), mainly among PLWHA and women patients. PLWHA go to healers when facing limited solutions in health system. There are fewer associations for TB patients, and less education and sensitization sessions to give them opportunity for sharing disease status and learning from other TB patients. TB patients and PLWHA still need to better understand their disease and its implication. Access to care (diagnosis and treatment) remains one of the key issues in health system, especially for PLWHA. Individual counseling is centered among PLWHA but not for TB patients. With research progress and experiences sharing, TB patients and PLWHA have some hope to implement their life project, and to receive psychosocial and nutritional support.

**Conclusion:**

Despite international aid, TB patients and PLWHA are facing misconceptions effects. There is a need to reinforce health education towards patients and healers, inside community, health centers and associations, and for specific settings. International aid must be adapted to specific targets and strategies implementing programs. Maintaining psychosocial and nutritional support is crucial for better outcomes of medication adherence. Individual counseling has to be centered among TB patients and PLWHA.

## Background

Tuberculosis (TB) and human immunodeficiency virus (HIV) infection remain among public health priorities throughout the world. TB prevalence rate was 397 (179–654) per 100,000 populations with 20 % of cases tested HIV positive in 2009 [1]. The prevalence of HIV among adults aged 15–49 years in Burkina Faso was 2.1 % in 2001 and 1.6 % in 2007 [2].

Facing high rates of TB and HIV infection, Burkina Faso receives different aid from international (bilateral and multilateral) levels. Indeed, the financing of TB (90–100 %) [3, 4] and HIV (62.6 %) [4] programs are primarily from international level (from bilateral and multilateral cooperation, international non-governmental organizations-NGOs mainly Global Found, and international foundations and firms). Despite the efforts from national and international levels, new TB cases are growing to 2660 in 2006 at 3041 cases 2010 [1, 5]. The percentage of TB treatment failure is growing with 7.0 % in 2007 and 8.5 % in 2009 [1, 5] with an estimated multidrug resistance cases to anti-tuberculosis drugs (MDR-TB) 2009 among new pulmonary TB cases notified of 34 (0–92) and among retreated pulmonary cases notified of 88 (0–230) [1]. The percentage of population in need of treatment with access to antiretroviral drugs in Burkina Faso was 60 % in 2009 [2]. There is a need to deeply understand the specific context and needs about TB and HIV infection.

Misconceptions exist related to the knowledge, attitude, perception and practices about TB at the individual and community level [6–13]. The consequences of the misconceptions are discrimination and stigmatization and the interpretations of the illness and the wellness about TB and HIV infection in rural and urban community. Additionally, this can lead to lack of compliance in continuing treatment [14] resulting in drug resistance. The percentages of confirmed TB multi-drug resistance (MDR-TB) among the TB cases tested for MDR-TB in 2009 and 2010 are growing with 34 and 39 % respectively [1].

Women facing TB infection fear more the psychosocial consequences and their social isolation [15, 16] than the actual disease itself. Issues related especially to TB infection, additional problems include the TB case management (the organization around the case diagnosis, the treatment, and the care), the characteristics of the patient, the patient in his community and his family, the support received, the geographical access to care, the poverty, and the experience about the use of health services [17]. Moreover many HIV-infected patients do not understand the importance of the diagnosis and may look for alternative care, such as consulting village healers [18].

The purpose of this study is to report the current situation of TB patients and patients living with HIV/AIDS (PLWHA) facing their disease and its implications in Burkina Faso.

## Methods

### Study setting

This cross sectional study was undertaken in health centers and NGOs located in the *Centre* and *Hauts*-*Bassins* regions of Burkina Faso. These two regions represent about 40 % of the total annual TB cases nationwide, have the highest HIV prevalence and have the largest number of NGOs providing antiretroviral treatment (ART) in the country Ref. [5, 19].

### Study design

Cross sectional study using mixed method (qualitative and quantitative) was used; TB and HIV co-infected patients were excluded. The qualitative study was been conducted before the quantitative one; it highlighted the attitudes and opinions of the interviewees. The quantitative study measured the amount of interviewees who had a certain behavior.

#### The qualitative study design

Planned for the period from 2nd to 10th July 2010, a total of 16 focus groups were held with 6 persons per group. Patients were selected in *Hauts*-*Bassins* region, both from rural area (Orodara Health District) and urban area (Bobo Dioulasso). These patients were not involved in design of the quantitative study. Patients were stratified into groups by setting and by gender. The 16 focus groups included 2 TB female and 2 TB male groups from the rural area; 2 TB female and 2 TB male groups from urban area; 2 patients living with HIV/AIDS (PLWHA) male and 2 PLWHA female groups from rural area; 2 PLWHA male and 2 PLWHA female groups in urban area. The group members were recruited with the help of health workers who informed the patients and negotiated the appointment time. Another appointment was negotiated for the focus group. The place of the focus group was in the TB detection and treatment Center (TDTC) for the TB patients and the NGOs for the PLWHA. The focus group were conducted to emphasize relevant questions related to attitude, beliefs, stigmatization, relation patient-to-patient, community support. PLWHA with TB co-infection were excluded.

A standardized list of open-ended questions was used for discussions. This list was created by using themes retained for the discussions. Some of these themes derived from individuals interviews we undertake prior the focus group discussions. Some of the themes used are listed below: patient reaction to disease diagnosis, perception of the disease (self-esteem, quality of life, contamination by disease, prevention of the disease, hope of the patients, etc.); the life experiences and the sharing of the disease status; the aspects related to gender; the behavior patient-others-patient; the observance of the treatment (difficulties, constraints, life experiences, etc.); diagnosis and treatment costs; changing needs due to the disease (family support/aid, participation in social activities, work status, etc.); and the relationships and the future of the patient.

Discussion on a specific topic area continued until no new information was generated. Group discussions were tape-recorded and transcribed, and themes were identified through independent content analysis of the transcriptions and assistant moderator notes.

One moderator with great experience in focus groups, facilitated all the discussions undertaken in local language (*Dioula*) or in *French*. He was assisted by a nurse also fluent in *Dioula* and French. We used a focus group guide, ensured consistent data collection across groups and provided flexibility to obtain clarification [20, 21].

To minimize ‘social desirability’ [22], participants were reminded that the facilitators had no link with the health department. They were not working in the health centers and associations-NGOs concerned by the survey.

Focus groups were audio recorded and transcribed verbatim for analysis using a computer-based qualitative software program (QSR NVIVO 2.0). All French transcripts were translated in English by the moderator to ensure accuracy.

Content analysis was run by examining the major themes and patterns that emerged from the data, using ‘Concept Analysis’, an inductive approach for constructing and confirming theory through systematic data coding [23].

To control for subjectivity during analysis and ensure inter-coder agreement, two researchers independently coded the data and then the two researchers compared the results. Inconsistencies and disagreements in coding were discussed and resolved before final analysis.

#### The quantitative study design

In the *Centre* region, TB patients were identified from data provided by the National TB Diagnosis, Treatment and Research Centre, and by the Health Districts (HDs), of Boulmiougou, Baskuy, Sig-Noghin, Bogodogo, and Nongr-Massom. In the *Hauts*-*Bassins* region, TB patients were identified from data provided by the Regional TDTC, Souro Sanou National Teaching Hospital (SSNTH) and by theHDs of Orodara, Do, Dafra, Lena, Houndé, and Karangasso-Vigué.

PLWHA patients were identified from data provided by various NGOs and HDs, which were the same as those for the TB patients. The NGOs were the Association-Espoir-Vie (AES) and Responsabilité-Espoir-Vie-Solidarité (REVS+) from Bobo Dioulasso in the *Hauts*-*Bassins* region and the Association des Jeunes pour la Promotion des Orphelins (AJPO) from Ouagadougou in the *Centre* region. HIV and TB statuses were confirmed from the medical records of each patient.

The sample size was calculated using the online sample size calculation software provided by RASOFT [24]. A common margin of 5 % was used. Thus a confidence level of 95 % was chosen, with a response distribution of 50 % and a power of 80 %. The distribution was assumed to be normal and, knowing that the total TB cases for the regions were 1832 in 2008 [5, 19], the smallest TB sample size expected was 316 TB cases for the two regions. In terms of HIV sample size, the normal rule of having one case for at least two controls was applied, thus at least 600 HIV cases were needed.

This study has been planned for the period from 1st to 30th August 2010 after advice and guidance were obtained from the National TB program of Burkina Faso, together with expert opinion from the West African Health Organization.

A consecutive (successive) method was used for patient recruitment. The inclusion criteria were met when the patient was a confirmed TB case from a TB clinic and was undergoing anti-tuberculosis treatment. Similar criteria were used for HIV cases from AIDS clinics under HAART. The patients were required to be 15 years or older and living in the study setting. Cases from both sexes were included. After the cases were identified, this study included two profiles of patients: HIV infected patients and TB patients only. The co-infected patients for TB and HIV are excluded.

After obtaining informed consent from each patient, a face to face interview was conducted using a semi-structured questionnaire. For that, we used the information gathered by qualitative study to elaborate the semi-structured questionnaire consisting of two parts: the socio-demographic information of the patient and general questions about the self and social experience of the disease: adapted attitude index [25], adapted perception index (discrimination, stigma and isolation) [26], and awareness of disease transmission. Additional information was gathered about psychosocial and behavioral information.

The interviewer recorded and completed the individual’s information during the interview (15–20 min). The remaining section of the questionnaire lasted approximately 25–30 min.

Before conducting the final survey, the questionnaire underwent pre-testing in order to reduce bias and to better control the time needed to complete the questionnaire.

The data was entered into EPIDATA and analyzed using the SPSS PC statistical package, version 18.0. The cut-off point for continuous variables was the median. The level of significance was 0.05. A comparison of the variables, between the HIV only infected patients and those having tuberculosis, was carried out using the Chi square test for categorical data and *t* test for continuous data. The p value is identified significance between TB and HIV patients.

#### The mixed methods design strategies

An initial phase of qualitative data collection and analysis has been conducted and followed by a phase of quantitative data collection and analysis: sequential exploratory design. The purpose was to specify and quantify findings from other methods.

From the attributes used for the qualitative study, the same were used as the guidance for the redaction both qualitative and quantitative studies matching the trend of the results. The data were from the following themes or attributes: reactions to disease diagnosis, perception of the disease, experiences lived with the relatives of the patient and his community about the disease, aspects related to gender, sharing of the disease status and behavior patient-others-patient, observance of the treatment and costs of diagnosis and treatment, and patient needs and perspectives.

Both results have been presented using ad-hoc approach.

### Research approval and ethics

The present study was approved by the Research Ethics Committee of Burkina Faso and the Ministry of Health in July 2010. These approvals contributed a lot for the carrying out of the study in the Target regions.

Informed consent of the interviewees was sought and granted. Also, participants’ anonymity and confidentiality were ensured.

## Results

### Participants and samples characteristics

The qualitative study has been conducted from 7th to 14th July 2010. Table 1 shows the characteristics of the 96 participants recruited to the qualitative design study.

**Table 1:** Characteristics of study participants in the qualitative study

Type of participants	Focus group discussions (n = 16)	Gender	Number of patients (n = 96)	Zones represented	Age [mean (range)]
TB	2	Male	12	Rural	34 (16–52)
	2	Male	12	Urban	35 (17–56)
	2	Female	12	Rural	32 (15–49)
	2	Female	12	Urban	33 (16–50)
Patients living with HIV (PLWHA)	2	Female	12	Rural	34 (17–58)
	2	Female	12	Urban	34 (18–55)
	2	Male	12	Rural	35 (16–54)
	2	Male	12	Urban	37 (17–57)

About the quantitative study conducted from 1st August to 8th October 2010, a total of 862 patients, including 309 (35.8 %) TB patients and 553 (64.2 %) PLWHA participated in this study. 52.0 % were from *Hauts*-*Bassins* region and 48.0 % from *Centre* region. There was no difference statistics between both *Centre* and *Hauts*-*Bassins* settings; even the *Hauts*-*Bassins* region is more rural than the *Centre* region. In brief, the mean age of the PLWHAs was 36.5 ± 10.6 years old, and 57.1 % were female gender (see Table 2 for more details).

**Table 2:** Socio-demographic variables, association member status and cost of diagnosis and treatment by patient profile

Items	TB (n = 309)	HIV (n = 553)	Total (n = 862)	p*
*Region*				
Hauts-Bassins	150 (48.5)	298 (53.9)	448 (52.0)	0.151
Centre	159 (51.5)	255 (46.1)	414 (48.0)	
*Area*				
Rural	227 (73.5)	149 (26.9)	376 (43.6)	<0.001
Urban	82 (26.5)	404 (73.1)	486 (56.4)	
*Sex*				
Female	87 (28.2)	405 (73.2)	492 (57.1)	<0.001
Male	222 (71.8)	148 (26.8)	370 (42.9)	
*Age group*				
<36.5 years old	203 (65.7)	280 (50.6)	483 (56.0)	<0.001
≥36.5 years old	106 (34.3)	273 (49.4)	379 (44.0)	
*Education level*				
No educated	144 (46.6)	270 (48.8)	414 (48.0)	0.579
Educated	165 (53.4)	283 (51.2)	448 (52.0)	
*Profession*				
Others	238 (77.0)	490 (88.6)	728 (84.5)	<0.001
Private and public sector	71 (23.0)	63 (11.4)	134 (15.5)	
*Member of health mutual*				
No	305 (98.7)	534 (96.6)	839 (97.3)	0.099
Yes	4 (1.3)	19 (3.4)	23 (2.7)	
*Member of association since you know your disease status*				
No	286 (92.6)	263 (47.6)	549 (63.7)	<0.001
Yes	23 (7.4)	290 (52.4)	313 (36.3)	
*First choice for consultation when sick*				
Healers	47 (15.2)	118 (21.3)	165 (19.1)	0.038
Clinics	262 (84.8)	435 (78.7)	697 (80.9)	
*How expensive do you think TB diagnosis and treatment is in your country?*				
Free of charge	175 (56.6)	0 (0.0)	175 (20.3)	<0.001
Reasonably priced	47 (15.2)	27 (4.9)	74 (8.6)	
Somewhat/moderately expensive	63 (20.4)	204 (36.9)	267 (31.0)	
Very expensive	24 (7.8)	322 (58.2)	346 (40.1)	

* The p values were performed based on the calculation of the Chi square test

### Triangulation of results from both quantitative and qualitative study

For reactions facing to disease diagnosis, perception of the disease and experiences lived with the relatives of the patient and his community about the disease, patients from both quantitative and qualitative study perceived as grave diseases and faced stigma, perceived bad attitude towards TB/HIV and were discriminated against or isolated. Related to gender, women were exposed to social and financial problems. HIV groups participated in education and sensitization sessions related to activities from associations-NGOs than TB patients. For both TB and HIV patients, treatment emerged as key challenge for patients. Related to their needs and perspectives, PLWHA were more expressed than TB patients and support can be encouragement, advice, psychosocial support, food, health education, and home visits, was really helpful. All patients still hoped to receive support primarily in the form of medicines and supplemental nutrition.

### Reactions to disease diagnosis, perception of the disease

All patients were shocked hearing about having TB or HIV. TB and HIV, locally called “*white cough*” and “*spider*”, are still perceived as grave diseases. At the announcement of their status, all patients felt bad and associated the diagnosis to a death sentence. Moreover, they expressed fear because they thought about the interpretation of the disease in the community: “*having TB means you may have HIV, and* vice versa”. Indeed, 30.6 % of TB and PLWHA patients from the total sample size feared that other persons would know their disease status, especially about TB patients (37.5 %) as shown in Table 4. TB and PLWHA patients faced stigma, perceived bad attitude towards TB/HIV and were discriminated or isolated (Table 4). For TB and PLWHA patients in focus group, some symptoms such as losing weight, fatigue and fever were perceived to indicate the existence of one of both TB and HIV infections. Some people with TB and HIV infections had misconceptions of the way in which they can transmitted the disease, for example, by simple contact, or from eating from the same plate or utensils. The gravity of the HIV infection is greater expressed by PLWHA patients who seek care with healers, as confirmed by 21.3 % of PLWHA patients (Table 2); because they think that medical facilities do not have solution for them.

### Experiences lived with the relatives of the patient and his community about the disease

Across all focus groups, TB patients and PLWHA felt that their friends and relatives distanced themselves after learning their status. They still perceived stigmatization and some (more related to PLWHA) lost their home and job because of their status. Indeed, about 93 (10.8 %) patients had to change their housing after contracting their illness. Indeed, up to 12.7 % of PLWHA patients pointed out this fact (Table 3). There was a proportional difference between patient groups (p = 0.018). Unfortunately, 131(14.7 %) patients revealed that they had lost their job following their disease diagnosis (Table 3); there was no proportion difference between patient groups (p = 0.484). Up to 683 (79.2 %) of TB and PLWHA hid their disease status from others (Table 3); among the 683 TB patients and PLWHA who revealed their status, 19.5 % informed their partners, 32.5 % informed family members, 15.4 % informed health workers, and 23.7 % informed a staff of the association. Furthermore, 668 (77.5 %) patients felt that the relatives and friends talked about them when they were not present (Table 3). At least 124 (14.4 %) patients said that they stayed away from people in order to avoid rejection (Table 3); There was proportion difference between patient groups (p < 0.001). Some patients recognized that not all people behaved this way, as revealed by focus groups. To avoid being discovered as TB patients, some of the TB patients, mainly from rural setting, preferred to go outside their community for treatment because under DOTS, they will be identified as TB patients by the daily appointments at the health centers. Indeed 737 (85.5 %) patients agreed that they were bothered by others because they are TB patients or PLWHA; and 58.2 % of PLWHA patients (were more concerned by this issue (Table 4). There was proportion difference between patient groups (p < 0.001).

**Table 3:** Description of the variables related to psycho-social and behavioral variables by patient profile

Items	TB (n = 309)	HIV (n = 553)	Total (n = 862)	p*
*Did your housing change since you know that you are sick?*				
No	286 (92.6)	483 (87.3)	769 (89.2)	0.018
Yes	23 (7.4)	70 (12.7)	93 (10.8)	
*Do you lose your job because you are TB or HIV patient?*				
No	270 (87.4)	492 (89.0)	762 (85.3)	0.099
Yes	39 (12.6)	61 (11.0)	131 (14.7)	
*Do you stay away from people in order to avoid rejection?*				
Totally agree	14 (4.5)	2 (0.4)	16 (1.9)	<0.001
Rather agree	53 (17.2)	55 (9.9)	108 (12.5)	
Neutral	75 (24.3)	262 (47.4)	337 (39.1)	
Rather disagree	78 (25.2)	232 (42.0)	310 (35.9)	
Totally disagree	89 (28.8)	2 (0.4)	91 (10.6)	
*Do you think that you will share you TB or HIV disease experience with others patients?*				
Totally agree	147 (47.6)	8 (1.4)	155 (18.0)	<0.001
Rather agree	94 (30.4)	200 (36.2)	294 (34.1)	
Neutral	44 (14.2)	87 (15.7)	131 (15.2)	
Rather disagree	21 (6.8)	233 (42.1)	254 (29.5)	
Totally disagree	3 (1.0)	25 (4.5)	42 (3.2)	
*Do you ask your partner to use the condoms during the sexual intercourses?*				
Never	171 (55.3)	309 (55.9)	480 (55.7)	0.005
Almost never	27 (8.7)	15 (2.7)	42 (4.9)	
Sometimes	44 (14.2)	36 (6.5)	80 (9.3)	
Often	21 (6.8)	22 (4.0)	43 (5.0)	
All the time	46 (14.9)	171 (30.9)	217 (25.2)	
*Do you tell to someone something about your TB status?*				
No	24 (7.8)	155 (28.0)	179 (20.8)	<0.001
Yes	285 (92.2)	398 (72.0)	683 (79.2)	
*Do you think that one says something about your status?*				
Nothing	15 (4.9)	87 (15.7)	102 (11.8)	<0.001
Neutral	58 (18.8)	34 (6.1)	92 (10.7)	
Something	236 (76.4)	432 (78.1)	668 (77.5)	
*Do you think to receive the support from others?*				
Not at all	32 (10.4)	60 (10.8)	92 (10.7)	<0.001
A little	102 (33.0)	111 (20.1)	213 (24.7)	
A moderate amount	73 (23.6)	134 (24.2)	207 (24.0)	
Very much	95 (30.7)	218 (39.4)	313 (36.3)	
An extreme amount	7 (2.3)	30 (5.4)	37 (4.3)	

* The p values were performed based on the calculation of the Chi square test for categorical data

**Table 4:** Comparison of psycho-social and behavioral aspects between TB and HIV patients

Items	TB (n = 309)	HIV (n = 553)	Total (n = 862)	p*
*Are you afraid to transmit your disease to others?*				
Not at all	14 (4.5)	0 (0.0)	14 (1.6)	<0.001
Neutral	53 (17.2)	92 (16.6)	145 (16.8)	
A bit afraid	38 (12.3)	133 (24.1)	171 (19.8)	
Very afraid	137 (44.3)	241 (43.6)	378 (43.9)	
Extremely afraid	67 (21.7)	87 (15.7)	154 (17.9)	
*Do you (your-self) regularly use condoms (female for female and male for male)?*				
Never	173 (56.0)	310 (56.1)	483 (56.0)	0.003
Almost never	29 (9.4)	16 (2.9)	45 (5.2)	
Sometimes	48 (15.5)	45 (8.1)	93 (10.8)	
Often	17 (5.5)	22 (4.0)	39 (4.5)	
All the time	42 (13.6)	160 (28.9)	202 (23.4)	
*Do you fear that other persons know your disease status?*				
Totally disagree	75 (24.3)	106 (19.2)	181 (21.0)	<0.001
Rather disagree	49 (15.9)	140 (25.3)	189 (21.9)	
Neutral	69 (22.3)	199 (36.0)	268 (31.1)	
Rather agree	74 (23.9)	74 (13.4)	148 (17.2)	
Totally agree	42 (13.6)	34 (6.1)	76 (8.8)	
*Do you think that you will share your disease experience with others patients?*				
Totally disagree	147 (47.6)	233 (42.1)	380 (44.1)	<0.001
Rather disagree	94 (30.4)	200 (36.2)	294 (34.1)	
Neutral	44 (14.2)	87 (15.7)	131 (15.2)	
Rather agree	21 (6.8)	8 (1.4)	29 (3.4)	
Totally agree	3 (1.0)	25 (4.5)	28 (3.2)	
*Are you bothered because you are TB patient?*				
Never	74 (23.9)	51 (9.2)	125 (14.5)	<0.001
Sometimes	68 (22.0)	132 (23.9)	200 (23.2)	
Often	92 (29.8)	100 (18.1)	192 (22.3)	
All the time	75 (24.3)	270 (48.8)	345 (40.0)	
*Are TB and HIV patients isolated by their community?*				
Yes	102 (33.0)	290 (52.4)	392 (45.5)	<0.001
No	207 (67.0)	263 (47.6)	470 (54.5)	
*Are TB and HIV patients isolated by their family?*				
Yes	101 (32.7)	358 (64.7)	459 (53.2)	<0.001
No	208 (67.3)	195 (35.3)	403 (46.8)	
*Are TB and HIV female patients isolated by their family?*				
Yes	92 (29.8)	236 (42.7)	328 (38.1)	<0.001
No	217 (70.2)	317 (57.3)	534 (61.9)	
Attitude index (mean ± SD)	14.0 ± 1.8	9.4 ± 4.2	11.0 ± 4.1	<0.001
Discrimination and isolation index (mean ± SD)	4.2 ± 2.1	3.8 ± 1.6	3.9 ± 1.8	0.006
Stigma (mean ± SD)	9.4 ± 2.5	6.3 ± 1.9	7.4 ± 2.6	<0.001

*SD* standard deviation

* The p values were performed based on the calculation of the Chi square test for categorical data and t test for continuous data

Although all patients reported that they received information on how to avoid transmitting the disease to others; only 703 (81.6 %) patients were afraid of spreading the disease to others (Table 4). Indeed, up to 483 (56.0 %) patients never used condoms (Table 4). In addition, 480 (55.7 %) patients never asked their partners to use condoms during sexual intercourse (Table 3). And, all patients had on average, more than one sexual partner.

### Aspects related to gender

Participants reported stereotypical gender roles and still perceived stigmatization but less discrimination. Related to gender aspects, women remained financially dependent on their husbands and were subsequently exposed to social and financial problems when having TB or HIV infection. Women said they were abandoned by their husbands. Worse, they were more marginalized when their husbands were suspected of having died of HIV. Widowed women with HIV, even those abandoned by their husbands, faced emotional and economic burdens of the entire family. They had to take care of children and household, and some times of the husband when he was sick (from TB or HIV infection). Men generally hid their status from their wives. Moreover, women complained that when they were infected by TB or HIV, their husbands did not take care of them. Rather, husbands could be remarried when they were not sick.

From the quantitative data, TB patients and PLWHA perceived isolation by their community (45.5 %) and from their family (53.2 %), as showed in Table 4; in addition, patient isolation was identified as an important issue for women (38.1 %), but less so for men. The issue of isolation was more emphasized by PLWHA in community, in family and related to female gender.

### Sharing of the disease status and behavior patient-others-patient

TB patients rarely shared their status or talked to other TB patients. Indeed as TB patients said, there were less associations-NGOS for them, and less education and sensitization sessions to give them opportunity to share their disease status and to learn from other TB patients. It was more common to hear from HIV groups that they participated in education and sensitization sessions related to activities from associations-NGOs.

In the perspective of sharing TB or HIV disease experience with others patients, about 440 (52.1 %) patients said they would do it (Table 3).

### Observance of the treatment and costs of diagnosis and treatment

Across the discussions, treatment emerged as key challenge for TB patient. Even if the treatment is free of charge, there is other problems: the long length of the treatment, the daily geographical access to health center, the quantity and the size of the medicines, the injections for those under retreatment, the need to not take breakfast before taking medicines, side effects of medicines, and the need to eat well 1 h after treatment. For PLWHA, they got aid from associations-NGOs for the treatment and some medical and biological examinations.

Patients were interviewed about the reasons why patients would stop receiving the treatment. Reasons were: considering that patient was cured even if the treatment was not finished (48.7 %); stigmatization by entourage (46.3 %); having a problem of transportation (38.5 %); difficulty of going daily to the treatment center (44.7 %); financial problems (24.6 %). Other reasons (29.1 %) were: physical weakness of the patient and needing help to go to health center, raining period/season, long distance and remote health area, beliefs, insufficient information and ignorance, travel, forgetting, too much pills to swallow, unwillingness to go to the health center.

About the costs of diagnosis and treatment, the data from quantitative study showed that 56.6 % of TB patients think it is free of charge whilst that is not at all free for PLWHA (Table 2). Indeed 95.1 % of PLWHA said that the costs were expensive (Table 2).

### Patient needs and perspectives

All patients indicated that at the announcement of their results, they were very concerned about the future but, they were strongly encouraged by health workers (especially for TB patients) and counselors from associations-NGOs (for PLWHA); they improved their mood or outlook.

Related to social support and perspectives, only 2.7 % of the patients were health insurance members (Table 2). 36.3 % of the patients were association’ members since they know their disease status (Table 2); that was more the case for PLWHA (52.4 %) and fewer for TB patients (7.4 %).

About the question “*Do you think you need to receive support from others*?”, 350 (40.6 %) of TB patients and PLWHA needed it (Table 3). About the question “*From whom do you receive support*?”, TB patients pointed out the support from relatives and family members (51.9 %) and government through health facilities (67.6 %). PLWHA received support from association or NGOs (51.3 %) and relatives and family members (51.9 %). The support from the patient’s workplace was very low (4.9 %).

During the focus group discussions, PLWHA expressed that support from associations-NGOs, such as encouragement, advice, psychosocial support, food, health education, and home visits, was really helpful.

PLWHA and TB patients stated that they feel hope for the future and have some ongoing projects. This positive outlook was based on the hope in research and the present medical therapy, particularly because the situation in the past was worse for PLWHA. All patients still hoped to receive support primarily in the form of medicines and supplemental nutrition.

## Discussions

This study shows that TB patients and PLWHA still faced many problems and difficulties (discrimination and stigmatization, need an access to disease diagnosis and treatment, need support from health workers and workers from associations-NGOs, etc.). Women with TB and HIV were especially vulnerable because they experienced additional emotional and financial problems.

Related to perceptions, a study showed that 54.8 % had negative attitudes and practices towards TB [6]. Misconceptions exist in such situation: patients stop treatment when the symptoms decrease [7] or when they feel better [8], about the necessity to separate the utensils used for eating in general population [9, 10] or hospitalization of patients [10], about the erroneous transmission routes such as blood and sexual fluids [9] and hereditary transmission [11]. For them, tuberculosis infection is incurable and dangerous [12, 13], transmittable and associated with HIV/AIDS leading to the understanding that TB is a very dangerous disease [13]. The consequences of the misconceptions are stigmatization and social isolation of TB patients and their families [12]. For example in the study from Shrestha-Kuwahara and colleagues, TB patients felt that “*Friends will run away from you*.” or “*They point to you with a finger and say that you have something ugly*” [22]. According to Baral and colleagues, the causes of discrimination by members of the general public were the fear of a perceived risk of infection: perceived links between TB and other causes of discrimination particularly poverty and low caste, perceived links between TB and disreputable behavior, and perceptions that TB was a divine punishment [27]. Related to TB and HIV infections, one study in Zambia showed that there is a new feature of stigma: a trigger for TB-HIV stigma [28]. That was the same feeling found in the present study and emphasized for PLWHA.

About the use of traditional healers services, about 60–80 % of African people rely on traditional healers for their health needs [29, 30]. Facing HIV infection, some of patients confided to the healers because they think that the health facilities cannot save them from death; that was the case in the present study. In Burkina Faso, a study showed strong correlation between consulting healers (first time being sick) and the region setting among HIV patients [18]. And about 15 % of traditional healers said they do not refer patients to health centers [31]. This shows the necessity to reinforce sensitization towards patients and healers related to HIV/AIDS for specific settings.

Related to gender aspects among TB patients, the findings of the present study were confirmed by Onifade et al. [32]. Indeed, the negative perceptions were the rejection and the burden on both sexes even if women reported feeling the burden of tuberculosis stigma more heavily than men [32]. Moreover according to Sudha and colleagues, men and children were perceived to get preferential attention by their families during illness [33]. For Weiss et al. [34] men frequently focused on financial concerns. For women, a diagnosis of TB can have serious repercussions for families and households [35].

Related to the observance of the treatment, Corless et al. found that treatment failure has been related to inappropriate regimens, the unavailability of drugs, or lack of access to health care [36, 37]. Moreover, the adverse effects of anti-tuberculosis drugs, sex and occupation are also predictive factors of successful treatment [38]. In addition to the importance of the regimens and access to drugs, the patients in the present study pointed out the importance of food. That was already emphasized by some studies which showed that the lack of food has also been noted to impact treatment adherence [37, 39].

Regarding the needs, the prior support needed was financial, transportation and follow by psychological, social, medical and physiological factors [40]. That was pointed out in the present study with additional point about food. Another important need was the opportunity for sharing experiences with other HIV patient groups so that patients could gain some hope from the associations and NGOs. Through associations and NGOs, PLWHA receive education and sensitization about their disease, and psycho-social support from other HIV patients with experience sharing. There is a need to re-organize health centers to consider this issue for TB patients. It is less the case for HIV patients for whom the guidelines foreseen individual counseling before, during and after the HIV test. TB and HIV patients face challenges regarding how to maintain the gains, especially considering the financial constraints of principal donor in the field who is unable to continue funding programs.

Even though these findings are relevant, despite attempts to reduce biases by excluding co-infected patients and follow rigorous methodology, the interpretations of these findings can be subject to criticisms linked to the nature of the study using focus group and cross sectional design. The stratification of the patients per group did not consider the status of the patient and its disease experience (example TB patient in retreatment). As observed by Shrestha-Kuwahara and colleagues, focus group methodology is inherently limited in its generalizability to broader populations and the conclusions drawn from this study apply only to the participating sites [22]; and the focus group method does not allow specific attribution of responses to specific respondents, thus barring quantification of formation, but may recruit those who were cooperative and willing to participate in the study.

## Conclusions

Burkina Faso receives different aid from international (bilateral and multilateral) level. It still remains that TB patients and PLWHA are subject to some key issues facing their disease: stigmatization due to misconceptions with its aftermaths such as rejection and emotional and financial problems. That was more emphasized about PLWHA and women patients. With the research progress and the experiences sharing, TB patients and PLWHA have some hope and would like to be able to implement their life project in future. There is a need to reinforce health education towards patients and healers, inside communities, in health centers and NGOs and associations, and for specific settings. The international aid can be used for adapting to specific targets for an effective fight against TB and HIV infection. The need is to address issues regarding patients who drop out of care when they are no longer symptomatic and patients not regularly taking their medication, resulting in drugs resistance and the spread of disease. This is one of the most important issues in controlling chronic communicable diseases such TB and HIV in the world.

In addition, it is crucial to maintain psychosocial and nutritional support to TB patients and PLWHA in order to reach better outcomes of the medication adherence and programs results. Individual counseling before, during and after testing has to be centered among patients infected with TB.

## Abbreviations

PLWHA: patients living with HIV/AIDS
NGO: Non-Governmental Organization
MDR-TB: tuberculosis multi-drug resistance
ART: anti retroviral treatment
TDTC: TB detection and treatment Center
NTDTRC: National TB Diagnosis, Treatment and Research Center
HD: health district
SSNTH: Souro Sanou National Teaching Hospital
AES: Association-Espoir-Vie
REVS+: Responsabilité-Espoir-Vie-Solidarité
AJPO: Association des Jeunes pour la Promotion des Orphelins
NTP: National TB program
WAHO: West African Health Organization
